# Antidepressants can help to prevent and manage long COVID depression, anxiety, brain fog and fatigue

**DOI:** 10.1007/s00406-024-01899-5

**Published:** 2024-09-26

**Authors:** Udo Bonnet, Jens Kuhn

**Affiliations:** 1 Department of Mental Health, Evangelisches Krankenhaus Castrop-Rauxel, Academic Teaching Hospital of the University of Duisburg, Essen, Castrop-Rauxel, Germany; 2 Department of Psychiatry and Psychotherapy, Faculty of Medicine, Landschaftsverband Rheinland-Hospital Essen, University of Duisburg-Essen, Essen, Germany; 3 Department of Psychiatry and Psychotherapy, University Hospital Cologne, Kerpener Str. 62, 50924 Cologne, Germany; 4 Alexianer Hospital Cologne, Alexianer Köln GmbH, 51149 Cologne, Germany

With great interest we read the article by Rosolen et al. in EAPCN, which describes that in an Italian case-control study, people who had contracted the SARS-COV-2 virus were more likely to be prescribed antidepressants (ADs) afterwards [1]. This was particularly the case in unvaccinated and elderly people, as well as in patients who had suffered a more severe COVID-19 [1]. These data support the finding that the COVID pandemic, but especially COVID-19 (i.e. the disease), promotes the development of anxiety and depression [2], provided that ADs are to be understood as a surrogate marker. Comparable data can also be found for other substance classes such as opioids and sleeping pills, which in turn could mean the (COVID-19) disease-related induction of sleep disorders and pain syndromes [3]. However, there is insufficient mention of the fact that there are hints that the depression-related risk appears to normalize again over time, whereas an increased risk of inducing psychotic disorders and dementia after COVID seems to remain [4].

Another point that needs to be mentioned in our opinion is that there is evidence that ADs can also be helpful in preventing severe COVID-19 and alleviating Long COVID symptoms such as depression, anxiety, cognitive dysfunction (“brain fog”) and fatigue [5, 6]. Specifically, for fluvoxamine there are currently at least 8 positive meta-analyses, 7 placebo-controlled randomized and 5 prospective studies that supported that this AD, when used for 10 to 14 days in addition to standard therapy for mild to moderate COVID-19, resulted in significantly fewer severe courses and deaths than placebo or standard therapy alone, with good tolerability [5]. Whether this applies to the entire class of AD substances warrants future well-controlled clinical studies on this issue. At least four large retrospective studies (each with more than 20,000 hospitalized patients) now indicate this [5]. Initial small clinical trials show relief of at least fatigue, cognitive dysfunctions and other psychiatric Long COVID symptoms with various ADs [5]. Not only fluvoxamine [7] but also the entire AD substance class are thought to have a pronounced protective (anti-inflammatory) effect against oxidative stress-mediated destruction in affected tissues that occurs following an excessive and prolonged inflammatory response („hyperinflammation“) to SARS-COV-2 virus entry into cells [5]. Furthermore, antiviral properties have been described for many ADs, including against SARS-COV-2 infections [5].

In addition to organic sequelae (e.g. pulmonary fibrosis, heart disease, strokes, kidney damage), the persistence of SARS-COV-2 viruses in immune-privileged regions of the body (e.g. eye, brain, testis), reactivation of other latent viruses, autoimmunity or misdirected immune responses to SARS-COV-2, persistent hyperinflammation with subsequent mitochondrial dysfunction, dysbiosis in the respiratory and gastrointestinal microbiome as well as protracted sickness behavior are discussed as biological mechanisms for Long COVID [5, 8]. Much of the neuropsychiatric symptoms of Long COVID, including depression, anxiety, fatigue and cognitive dysfunction, may also be promoted by premature (and hopefully reversible) “aging” of midbrain dopaminergic neurons [5, 8]. In line with (or in addition to) their general antidepressant, anxiolytic, anti-inflammatory and antiviral properties, ADs could be beneficial for Long COVID symptoms, as more than 95% of the 5-hydroxytryptamine (serotonin) in the body is localized in the gastrointestinal tract and SARS-COV-2-related dysbiosis of the microbiome there could promote and then maintain the development of a serotonin deficit, as recently found in the serum of affected individuals [6, 7]. This serotonin deficit could then be compensated by the serotonergic properties of ADs [6, 7] (which most ADs have, including ketamine and bupropion [5, 9]). In the context of the bio-psycho-social explanatory approach, it is becoming increasingly clear that psychosomatic and psychosocial factors can play a decisive symptom-maintaining role [5]. Good medical education about the potential adverse effects of AD, taking into account their placebo and nocebo properties [10], can also improve adherence and effectiveness of ADs in the COVID-19 and post-COVID population [5].

## References

[1] Rosolen V, Castriotta L, Driutti M et al (2024) Association between previous SARs-CoV-2 infection and new prescription of antidepressant drugs: a case-control study in Friuli Venezia Giulia region, Italy. Eur Arch Psychiatry Clin Neurosci. 10.1007/s00406-024-01846-4Epub ahead of print 38953980 10.1007/s00406-024-01846-4

[2] COVID-19 Mental Disorders Collaborators (2021) Global prevalence and burden of depressive and anxiety disorders in 204 countries and territories in 2020 due to the COVID-19 pandemic. Lancet 398(10312):1700–1712. 10.1016/S0140-6736(21)02143-7 34634250 10.1016/S0140-6736(21)02143-7 PMC8500697

[3] Xie Y, Xu E, Al-Aly Z (2022) Risks of mental health outcomes in people with covid-19: cohort study. BMJ 376:e068993. 10.1136/bmj-2021-068993 35172971 10.1136/bmj-2021-068993 PMC8847881

[4] Taquet M, Sillett R, Zhu L et al (2022) Neurological and psychiatric risk trajectories after SARS-CoV-2 infection: an analysis of 2-year retrospective cohort studies including 1 284 437 patients. Lancet Psychiatry 9(10):815–827. 10.1016/S2215-0366(22)00260-7 35987197 10.1016/S2215-0366(22)00260-7 PMC9385200

[5] Bonnet U, Juckel J (2024) The impact of antidepressants for COVID-19 and Post-acute COVID-19 syndrome. Fortschr Neurol Psychiatr. 10.1055/a-2374-2218. Epub ahead of print 10.1055/a-2374-2218 39313202

[6] Bonnet U, Kuhn J (2024) Serotonin deficiency and psychiatric long COVID: both caused specifically by the virus itself or an adaptive general stress response? Eur Arch Psychiatry Clin Neurosci. 10.1007/s00406-024-01769-0. Epub ahead of print 10.1007/s00406-024-01769-0 38413454

[7] Hashimoto K (2023) Detrimental effects of COVID-19 in the brain and therapeutic options for long COVID: the role of Epstein-Barr virus and the gut-brain axis. Mol Psychiatry 28(12):4968–4976. 10.1038/s41380-023-02161-5 37402856 10.1038/s41380-023-02161-5 PMC11041741

[8] Davis HE, McCorkell L, Vogel JM, et al. (2023). Long COVID: major findings, mechanisms and recommendations. Nat Rev Microbiol 21(3):133–146. doi: 10.1038/s41579-022-00846-2 10.1038/s41579-022-00846-2 PMC9839201 36639608

[9] Witt CE, Mena S, Holmes J, et al. (2023). Serotonin is a common thread linking different classes of antidepressants. Cell Chem Biol. 30(12): 1557–1570.e6. doi: 10.1016/j.chembiol.2023.10.009 10.1016/j.chembiol.2023.10.009 37992715

[10] Claus BB, Scherbaum N, Bonnet U. (2020). Effectiveness of an Adjunctive Psychotherapeutic Intervention Developed for Enhancing the Placebo Effect of Antidepressants Used within an Inpatient-Treatment Program of Major Depression: A Pragmatic Parallel-Group, Randomized Controlled Trial. Psychother Psychosom. 89(4): 258–260. doi: 10.1159/000505855 10.1159/000505855 32069467

